# Efficient conversion of biomass into lipids by using the simultaneous saccharification and enhanced lipid production process

**DOI:** 10.1186/1754-6834-6-36

**Published:** 2013-03-05

**Authors:** Zhiwei Gong, Hongwei Shen, Qian Wang, Xiaobing Yang, Haibo Xie, Zongbao K Zhao

**Affiliations:** 1 Division of Biotechnology, Dalian Institute of Chemical Physics, CAS, Dalian, 116023, PR China; 2 Dalian National Laboratory for Clean Energy, Dalian Institute of Chemical Physics, CAS, 457 Zhongshan Rd, Dalian, 116023, PR China; 3 University of Chinese Academy of Sciences, Beijing, 100049, PR China

**Keywords:** Microbial lipids, *Cryptococcus curvatus*, Biodiesel, Corn stover, Simultaneous saccharification and enhanced lipid production

## Abstract

**Background:**

Microbial lipid production by using lignocellulosic biomass as the feedstock holds a great promise for biodiesel production and biorefinery. This usually involves hydrolysis of biomass into sugar-rich hydrolysates, which are then used by oleaginous microorganisms as the carbon and energy sources to produce lipids. However, the costs of microbial lipids remain prohibitively high for commercialization. More efficient and integrated processes are pivotal for better techno-economics of microbial lipid technology.

**Results:**

Here we describe the simultaneous saccharification and enhanced lipid production (SSELP) process that is highly advantageous in terms of converting cellulosic materials into lipids, as it integrates cellulose biomass hydrolysis and lipid biosynthesis. Specifically, *Cryptococcus curvatus* cells prepared in a nutrient-rich medium were inoculated at high dosage for lipid production in biomass suspension in the presence of hydrolytic enzymes without auxiliary nutrients. When cellulose was loaded at 32.3 g/L, cellulose conversion, cell mass, lipid content and lipid coefficient reached 98.5%, 12.4 g/L, 59.9% and 204 mg/g, respectively. Lipid yields of the SSELP process were higher than those obtained by using the conventional process where cellulose was hydrolyzed separately. When ionic liquid pretreated corn stover was used, both cellulose and hemicellulose were consumed simultaneously. No xylose was accumulated over time, indicating that glucose effect was circumvented. The lipid yield reached 112 mg/g regenerated corn stover. This process could be performed without sterilization because of the absence of auxiliary nutrients for bacterial contamination.

**Conclusions:**

The SSELP process facilitates direct conversion of both cellulose and hemicellulose of lignocellulosic materials into microbial lipids. It greatly reduces time and capital costs while improves lipid coefficient. Optimization of the SSELP process at different levels should further improve the efficiency of microbial lipid technology, which in turn, promote the biotechnological production of fatty acid-derived products from lignocellulosic biomass.

## Background

Biodiesel is an important renewable fuel that is usually produced from vegetable oils or animal fats by transesterification with methanol/ethanol [1]. However, traditional oil-rich crops and plants are limited by land availability, and are in constant debate due to the food versus fuel issues. Thus, feedstock supply is the major obstacle for large-scale application of biodiesel. Alternative feedstocks for biodiesel have been pursued intensively in recent years. Oleaginous yeasts produce neutral lipids consisting of long-chain fatty acids comparable to those of conventional vegetable oils. Thus, microbial lipids have been suggested as a potential feedstock for biodiesel production [2,3]. However, the costs of microbial lipids remain prohibitively high for commercialization. To reduce the costs, it is important to explore sustainable raw materials such as lignocellulosic biomass and to develop integrated processes.

Carbohydrates derived from lignocellulosic materials have been used to culture oleaginous yeasts for microbial lipids [4-8]. In those cases, biomass was pretreated, hydrolyzed and the corresponding hydrolysates were used. There were also a few reports that integrated cellulose hydrolysis and lipid biosynthesis in a single bioreactor, where both cellulolytic enzymes and auxiliary nutrients were present. In one recent example of integration of lipid production and enzymatic hydrolysis of corn stover, lipid titre reached 3.23 g/L when the yeast *Trichosporon cutaneum* was cultivated in 50-L stirred-tank bioreactor [9]. In another example, lipid production by *Microsphaeropsis sp.* was carried out using steam-exploded wheat straw under solid-state culture conditions [10]. We recently reported the two-staged culture mode for lipid production, where cells were loaded in glucose solution without auxiliary nutrients [11,12]. Because cell density was high and cell propagation was inhibited by nutrient deficiency, lipid productivity and yield were significantly enhanced.

A number of oleaginous yeasts have been applied for lipid production [13]. *Cryptococcus curvatus* is an excellent lipid producer. It can use various cheap substrates such as biomass hydrolysates and has a good tolerance to major biomass-derived inhibitors [5,7,14]. In this paper, we describe the simultaneous saccharification and enhanced lipid production (SSELP) process that integrates the biomass hydrolysis step and an enhanced lipid accumulation step, to effectively convert lignocellulosic materials into lipids. Specifically, cells prepared in a nutrient-rich medium were inoculated at high dosage for lipid production in biomass suspension in the presence of hydrolytic enzymes without auxiliary nutrients. When corn stover regenerated from an ionic liquid-based pretreatment procedure was used, both cellulose and hemicellulose were consumed simultaneously. No xylose accumulation was observed, indicating that glucose effect was circumvented. The SSELP process offers high lipid coefficient, greatly reduces time and costs and appears promising for lipid production from lignocellulosic biomass.

## Results and discussion

### Effect of initial pH, enzyme dosages and temperature on the SSELP process

Lipid production by oleaginous microorganisms is usually considered as partially growth-coupled. In a typical batch culture, nutrient composition is designed to support cell propagation, and at the same time, to deplete the controlling nutrient for lipid accumulation [15]. While cell propagation and lipid accumulation are maintained in the same culture, neither of them is able to reach its maximal capacity. We recently showed that the two-staged mode was advantageous to achieve high productivity and yield for microbial lipid production [11,12]. In the first stage cells were cultivated in a nutrient-rich medium, while in the second stage cells were resuspended in a glucose solution without auxiliary nutrients. The two-staged culture mode provided a basic configuration to enhance lipid production. We categorized lipid production into six different culture modes mainly depending on substrates and auxiliary nutrients availability (Table 1). When cellulose is considered as the ultimate substrate, one can make hydrolysates first, followed by cultivation of oleaginous cells. Thus, the separated hydrolysis and lipid production (SHLP) process and the separated hydrolysis and enhanced lipid production (SHELP) process can be defined. Alternatively, one can integrate cellulose hydrolysis and lipid production in a single unit. Thus, the simultaneous saccharification and lipid production (SSLP) process and the SSELP process can be defined. We expect that the SSELP process should be much more efficient.

**Table 1:** Features of different culture modes used for lipid production

**Culture mode**	**Substrate**	**Auxiliary nutrients**	**Hydrolytic enzymes**	**Cell propagation**	**Lipid accumulation**
Single stage	Glucose	✓	×	✓	✓
Two-staged	Glucose	×	×	×	✓
SHLP ^*a*^	Cellulose hydrolysates	✓	×	✓	✓
SHELP ^*b*^	Cellulose hydrolysates	×	×	×	✓
SSLP ^*c*^	Cellulose	✓	✓	✓	✓
SSELP ^*d*^	Cellulose	×	✓	×	✓

^*a*^*SHLP*: Separated hydrolysis and lipid production; ^*b*^*SHELP*: Separated hydrolysis and enhanced lipid production; ^*c*^*SSLP*: Simultaneous saccharification and lipid production; ^*d*^*SSELP*: Simultaneous saccharification and enhanced lipid production.

**Figure 1:**
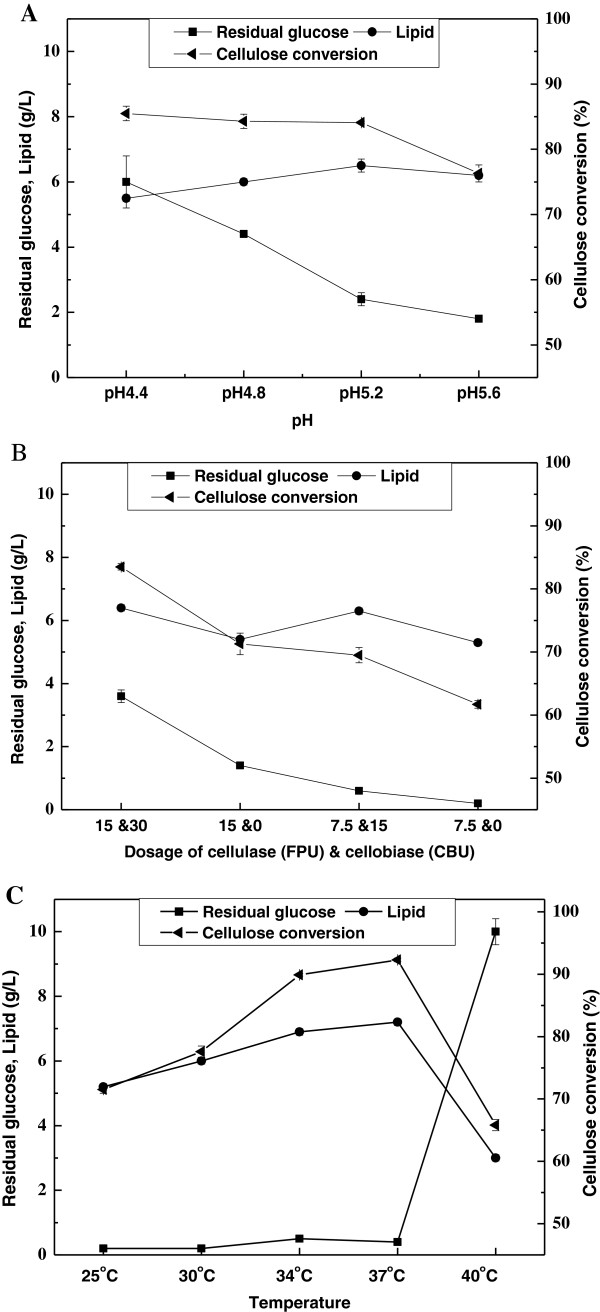
**Results of lipid production by *C. curvatus *using the SSELP process with different initial pH (A), enzyme dosage (B) and temperature (C).** Cellulose was loaded at 32.3 g/L, and the cultures were held for 48 h.

To optimize the operational parameters for the SSELP processes, a number of conditions were tested (Table 2). It was found that the highest lipid yield was obtained at initial pH 5.2 (Figure 1A). Cellulose conversion slightly decreased from 85.5% to 84.1% when initial pH increased from 4.4 to 5.2, while lipid titre increased from 5.5 g/L to 6.5 g/L. At pH 5.6, cellulose conversion and lipid titre decreased to 76.3% and 6.0 g/L, respectively. When initial pH increased from 4.4 to 5.6, residual glucose concentration decreased gradually, suggesting that cellulose hydrolysis became repressed. At pH 5.2, residual glucose concentration was 2.4 g/L, indicating cellulose hydrolysis and lipid production were well matched.

**Table 2:** Summary of experimental parameters tested for the SSELP process

**Parameters**	**Effect of initial pH**	**Effect of enzymes**	**Effect of temperature**
Initial pH	4.4, 4.8, 5.2, 5.6	5.2	5.2
Agitation speed (rpm)	200	200	200
Cellulase (FPU/g) & cellobiose (CBU/g)	15 & 30	15 &30, 15 & 0, 7.5 & 15, 7.5 & 0	7.5 & 15
Temperature (°C)	30	30	25, 30, 34, 37, 40

It was found that dosages of cellulase and cellobiase per gram cellulose at 15 FPU and 30 CBU, respectively, led to the highest lipid titre, cellulose conversion and residual glucose concentration (Figure 1B), which suggested that cell viability was the major constraint in the system. The lowest lipid titre and cellulose conversion were found when cellulase alone was used at 7.5 FPU/g cellulose. Lipid titre and cellulose conversion improved significantly when cellobiase was added. This was likely due to cellobiase-mediated clearance of cellobiose, which could inhibit cellulose hydrolysis [16]. Lipid titre increased little but cellulose conversion improved significantly when cellulase dosage was doubled. Since the costs of enzymes remain one of the major hurdles to the development of an economically viable cellulosic biofuel industry [17], a lower enzyme loading is more appealing. Thus, dosages of cellulase and cellobiase per gram cellulose at 7.5 FPU and 15 CBU, respectively, were chosen for further optimization.

Experiments were also done at different temperatures for 48 h with initial pH 5.2 (Table 2), and results are shown in Figure 1C. Lipid titre and cellulose conversion increased when the culture temperature increased from 25°C to 37°C, and residual glucose were all below 0.5 g/L. Lipid titre and cellulose conversion reached 7.2 g/L and 92.3%, respectively, at 37°C. However, when the experiment was done at 40°C, both lipid titre and cellulose conversion were significantly dropped, and residual glucose was 10.0 g/L, indicating that lipid biosynthesis was inhibited but cellulose hydrolysis was efficient at 40°C.

A major challenge for the traditional simultaneous saccharification and fermentation process is that saccharification and fermentation have different temperature optima [18,19]. Oleaginous yeasts often have optimal growth temperature around 30°C whereas enzymatic saccharification is optimal at about 50°C. Because *C. curvatus* is a mesophilic yeast, cell growth in YPD medium was significantly inhibited at 37°C (data not shown). However, *C. curvatus* cells were found highly active for lipid production at 37°C when the SSELP process was employed, as lipid titre reached the highest. This was likely due to the fact that the absence of auxiliary nutrients inhibited cell propagation and enabled lipid biosynthesis at a higher temperature. The shift of optimal temperature to 37°C was advantageous because enzymatic cellulose hydrolysis was faster.

### Time course of lipid production with different strategies

**Figure 2:**
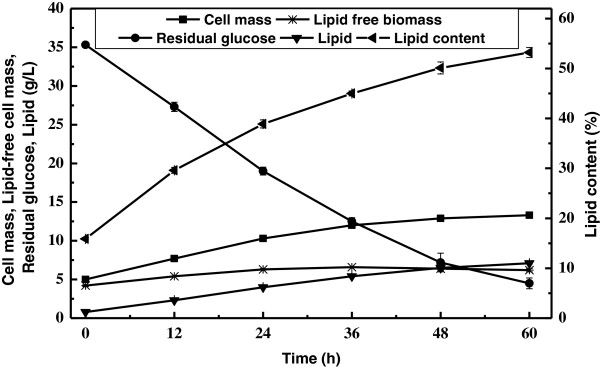
**Time course of lipid production by *C. curvatus *on glucose using the two-staged process.**

To test whether the two-staged culture mode was applicable for *C. curvatus*, inocula cells were resuspended in 35 g/L glucose solution and held at 30°C, 200 rpm for 60 h. Figure 2 showed the time course of the experiment. Glucose concentration was continuously dropping while cellular lipids and lipid content were increasing over time. Lipids increased linearly and lipid productivity reached 3.1 g/L/d during the first 36 h. Lipid-free cell mass increased only by 50% during the first 24 h and then kept constant, suggesting that cells were much more active for lipid biosynthesis rather than cell propagation. At the end of culture, residual sugar, cell mass, lipid content and lipid coefficient were 4.5 g/L, 13.3 g/L, 53.2% and 205 mg/g consumed glucose, respectively. Thus, the two-staged culture mode was efficient to convert glucose into lipids by *C. curvatus*. It should also be noted that sugar consumption rate of the two-staged culture mode was significantly higher than that of the single-staged mode [7].

**Figure 3:**
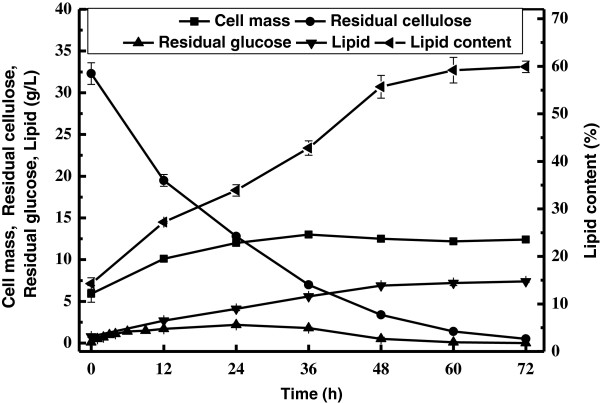
**Time course of lipid production by *C. curvatus *on cellulose using the SSELP process.** Experiments were done with 5 g/L CDW equivalent inocula, 32.3 g/L cellulose, 15 FPU cellulase and 30 CBU cellobiase.

Since our results demonstrated that cellulose hydrolysis and lipid production proceeded concurrently and efficiently during the SSELP process, we further followed the process for 72 h under an optimal condition (Figure 3). It was clear that cellulose concentration dropped and cellular lipid content increased over time. Lipid titre increased rapidly up to 48 h. Lipid biosynthesis slowed down from 48 h to 72 h, likely due to carbon source limitation. At the end of the culture, cellulose conversion, cell mass, lipid content and lipid coefficient were 98.5%, 12.4 g/L, 59.9% and 204 mg/g, respectively. The fact that glucose was at a low level during first 48 h suggested that enzymatic hydrolysis and lipid production were well coordinated. Glucose was undetectable after 60 h, indicating that hydrolysis became limiting.

**Figure 4:**
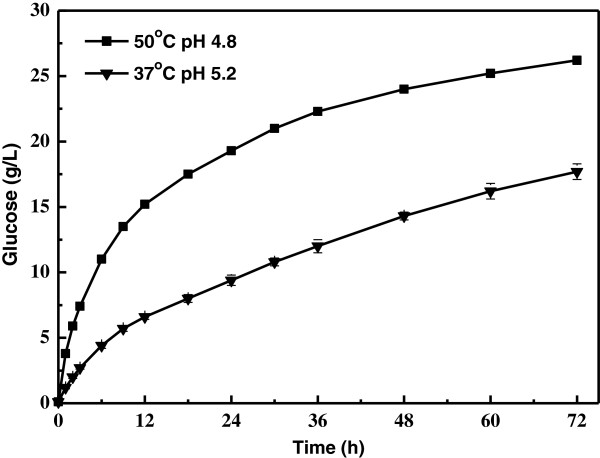
**Time course of enzymatic hydrolysis of cellulose.** Experiments were done with 32.3 g/L cellulose, 15 FPU cellulase and 30 CBU cellobiase.

Profiles of enzymatic hydrolysis of cellulose were shown in Figure 4. Glucose yield was only 44.3% after 72 h when the hydrolysis reaction was set at 37°C, pH 5.2. Even the reaction was run at optimal pH and temperature of 4.8 and 50°C, respectively, glucose yield was only 66.5%. In sharp contrast, cellulose conversion reached 98.5% with the SSELP process at 37°C, pH 5.2, in the presence of identical amounts of enzymes (Figure 3). The SSELP process essentially eliminated end-product inhibition for hydrolysis, because hydrolysis products were consumed by yeasts for lipid production. Thus, the SSELP process greatly reduced time when cellulose was used as the feedstock for lipid production.

Results shown in Figure 2 and Figure 3 were obtained with almost identical amounts of glucose equivalent carbon sources and initial inoculum sizes. Within 60 h, 30.8 g/L glucose was consumed in Figure 2, while 30.9 g/L cellulose was consumed in Figure 3. It was clear lipid titre data in Figure 3 were always higher than those in Figure 2 at given time points. For example, at 48 h, the SSELP process and the two-staged culture gave a lipid titre of 6.9 g/L and 6.5 g/L, respectively. The fact that glucose in the SSELP process was maintained at lower concentrations might avoid substrate inhibition. Moreover, the culture at 37°C should inhibit cell propagation but improve lipid biosynthesis than 30°C. By integration of cellulose hydrolysis and the two-staged lipid production into a single reactor, the SSELP process reduced the total time and capital costs, and improved efficiency for cellulose hydrolysis and lipid production. Similar results have been demonstrated for ethanol fermentation when lignocellulosic materials were used [20,21].

**Figure 5:**
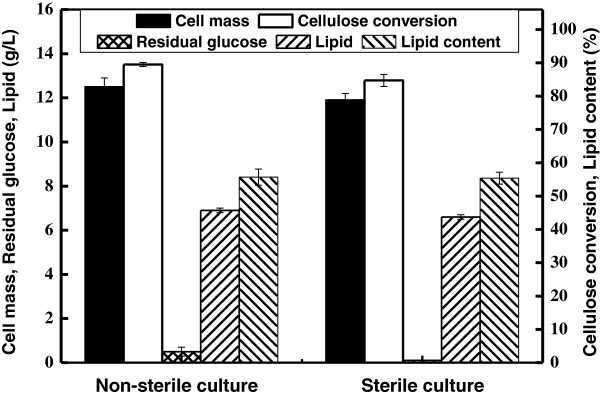
**Results of lipid production by *C. curvatus *using the SSELP process on cellulose with and without sterilization.**

Fast growth rate and tolerance low pH allow bacteria to effectively compete with yeasts. Bacterial contamination is one of the major challenges for yeast culture because it causes considerable economic losses by competing for substrates and inhibiting yeast cell growth [22]. For the SSELP process, no bacterial contamination was observed by microscope even though the system was not sterilized. Because the SSELP process was done in the absence of auxiliary nutrients and with large inoculum size, bacteria growth was effectively inhibited. When it was done without sterilization, cellulose conversion, cell mass and lipid content were 89.5%, 12.5 g/L and 55.7%, respectively (Figure 5). When the cellulose suspension was sterilized at 121°C for 18 min and all enzymes were sterilized by filtration using 0.22 μm membrane, cellulose conversion, cell mass and lipid content were 84.7%, 11.9 g/L and 55.4%, respectively. These data clearly demonstrated that slightly less lipids was produced when cellulose was sterilized. The fact that some inhibitory compounds such as furfural and hydroxymethylfurfural were present in the sterilized cellulose suspension might be responsible for such phenomena (Data not shown).

### Lipid production using regenerated corn stover as the feedstock

Both the SHELP process and the SSELP process were tested for lipid production using corn stover samples regenerated from the EmimOAc–NMP pretreatment procedure. When 5% (w/v) solid loading was used, lipid titre reached 6.2 g/L after 48 h, and lipid coefficient was 112 mg/g regenerated corn stover, or 81 mg/g raw corn stover (Table 3). The data were significantly higher than lipid titre of 3.23 g/L and lipid coefficient of 32.3 mg/g corn stover where an SSLP process was used for lipid production by the oleaginous yeast *Trichosporon cutaneum*[9]. Because auxiliary nutrients were absent for the SSELP process, lipid accumulation improved while cell propagation was inhibited. By controlling the availability of auxiliary nutrients, lipid production could be enhanced as indicated in our early study using glucose as the carbon source [11,12]. The lipid coefficient data were comparable to the results of solid-state fermentation for 10 days [10]. However, when *C. curvatus* cells were cultivated for the same time according to the SHELP strategy, lipid titre and lipid coefficient were 7.2 g/L and 138 mg/g regenerated corn stover, respectively. Lipid-free cell mass increased by 121%, which was even 52% higher than that obtained using glucose as the carbon source under two-staged culture conditions, suggesting that there were additional nutrients present in the corn stover hydrolysates. Although the SHELP strategy produced more lipids than the SSELP process, it required over 2-fold more enzymes and took extra time and space to prepare corn stover hydrolysates.

**Table 3:** **Results of lipid production by *C. curvatus *on corn stover regenerated from the EmimOAc–NMP system**

**Culture mode**	**Enzyme loading (/g corn stover)**	**Culture time (h)**	**Cell mass (g/L)**	**Lipids (g/L)**	**Lipid content (%)**	**Lipid coefficient (mg/g corn stover)**
SSELP	Cellulase: 4.0 FPU	48	-	6.0 ± 0.2	-	112 (81)^a^
	Cellobiase: 8.0 CBU					
	Xylanase: 5.0 mg					
SHELP	Cellulase: 10 FPU	48	16.5 ± 0.4	7.2 ± 0.2	43.4 ± 1.0	138 (100)^a^
	Cellobiase: 20 CBU					
	Xylanase: 10 mg					

^*a*^ Data in parenthesis are based on the mass of un-pretreated corn stover samples.

**Figure 6:**
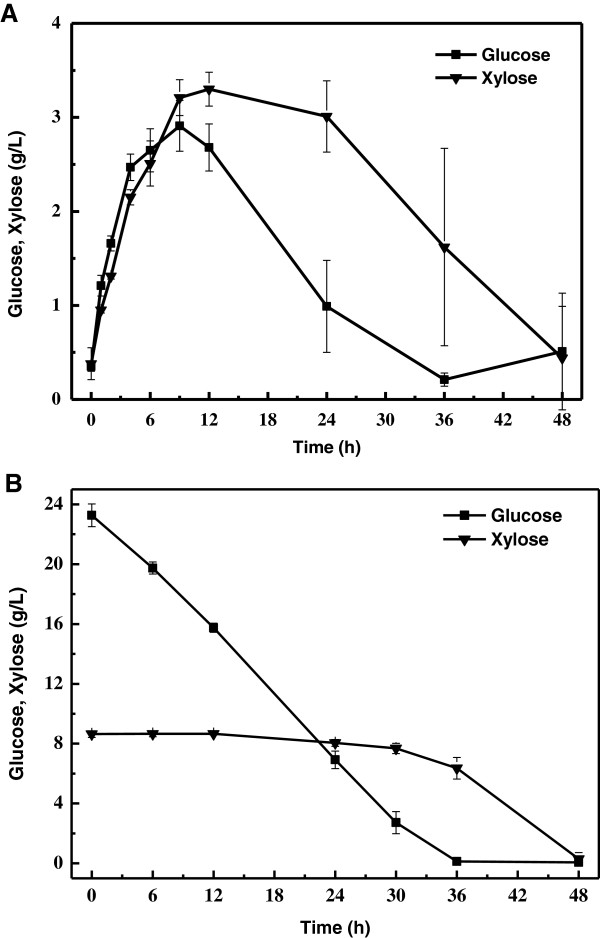
**Sugar consumption profiles by *C. curvatus *on corn stover regenerated from the EmimOAc–NMP system using the SSELP process (A) and the SHELP process (B).**

Biomass hydrolysates usually contain glucose, xylose, among other sugars. It is well known that most microorganisms prefer glucose over xylose and assume a stepwise sugar consumption profile because of glucose effect [23]. The fact that substrates are consumed stepwise usually leads to excess culture time and unstable kinetics. Thus, it is very important to develop strains or processes to ensure utilization of glucose and xylose simultaneously [24]. Figure 5 showed the sugar consumption profiles. When *C. curvatus* cells were cultivated in corn stover hydrolysates, glucose was consumed linearly from 0 to 24 h whereas xylose remained constant. Xylose consumption started until the glucose concentration dropped below 3.0 g/L (Figure 6B), indicating that *C. curvatus* strongly prefers glucose over xylose. In sharp contrast, when it was performed according to the SSELP process, glucose and xylose were both less than 3.5 g/L throughout the culture (Figure 6A). Glucose and xylose reached plateau concentrations at 9 h, and then were both consumed over time with no xylose accumulation, suggesting that both cellulose and hemicellulose were converted into lipids simultaneously. Thus, the SSELP process can offer a key advantage when lignocellulosic materials are used. It has been known that glucose repression can be circumvented if glucose concentration is low in a mixture of glucose and xylose [22,25].

### Fatty acid compositional profiles

Table 4 showed that lipid samples produced by *C. curvatus* according to the SSELP process contained mainly long chain fatty acids with 16 and 18 carbon atoms. The three major fatty acids were palmitic acid, stearic acid and oleic acid. The fatty acid compositional profiles were comparable to those of plant oil, suggesting that these products could be explored for biodiesel production [2,26]. The total contents of saturated fatty acids for some samples were close to 60%, suggesting that lipids from *C. curvatus* may also be explored as high value-added cocoa butter equivalents [27,28]. Interestingly, it seemed that lower culture temperatures favored saturated fatty acid (SFA) formation. This was consistent with our early study with this strain using N-acetyl-glucosamine as the substrate [29]. However, opposite trends were also found in the case of polyunsaturated fatty acids production by the filamentous fungus *Mortierella alpine*[30].

**Table 4:** **Fatty acid compositions of lipids from *C. curvatus *cultivated on cellulose according to the SSELP process**

**Entry**	**Culture temp. (**ºC**)**	**Myristic acid (%)**	**Palmitic acid (%)**	**Palmitoleic acid (%)**	**Stearic acid (%)**	**Oleic acid (%)**	**Linoleic acid (%)**	**SFA (%)**
1	25	1.5	36.4	0.6	21.3	38.4	1.6	59.3
2	30	1.4	39.0	-	21.2	37.9	0.5	61.6
3	34	1.4	36.2	-	20.0	41.3	1.0	57.7
4	37	1.3	33.8	-	20.7	43.2	0.9	55.9
5	40	1.1	28.8	0.2	21.8	46.7	1.6	51.6
6^a^	37	1.6	37.2	0.7	15.5	41.7	2.2	54.3
7^b^	-	-	26.0	-	34.4	34.8	3.0	60.4

^a^ EmimOAc–NMP regenerated corn stover was used. ^b^ Representative data for cocoa butter.

## Conclusions

We developed the SSELP process for efficient conversion of lignocellulosic materials into microbial lipids with high lipid coefficient. Because cells are inoculated in the auxiliary nutrient-free suspension of lignocellulosic materials containing hydrolytic enzymes, it can be performed without sterilization. It ensures efficient utilization of both cellulose and hemicellulose simultaneously, because glucose concentration remains low in the culture and glucose repression is circumvented. The SSELP process greatly reduces time and costs and appears promising for the production of fatty acid-derived products from lignocellulosic biomass.

## Methods

### Reagents and strain

Ionic liquid 1-ethyl-3-methylimidazolium acetate (EmimOAc) was supplied by Lanzhou Greenchem ILs, LIPC, CAS (Lanzhou, China) and used without further purification. Cellulose (sigmacell cellulose Type101, moisture content 10% (wt/wt)), cellulase and cellobiase were purchased from Sigma. The activities of cellulase and cellobiase were determined as 161.0 FPU/mL and 674.7 CBU/mL, respectively [31,32]. Xylanase was obtained from Imperial Jade Bio-Technology Co., Ltd. (Yinchuan, China), and the activity was 5000 kU/g. One unit of xylanase (U) was defined as the amount of enzyme which produces 1.0 μg of xylose from 1% xylan solution at pH 5.0, 50°C within 1 min. N-Methylpyrrolidone (NMP) and other reagents were analytical grade and purchased from local company.

The yeast *C. curvatus* ATCC 20509 was obtained from the American Type Culture Collection, and maintained at 4°C every two weeks on yeast peptone dextrose (YPD) agar slant (yeast extract 10 g/L, peptone 10 g/L, glucose 20 g/L, agar 15 g/L, pH 6.0).

### Pretreatment of corn stover with the EmimOAc–NMP solution

Corn stover harvested from countryside of Changchun, China, was milled to a particle size of 1–2 mm. The milled materials were washed to remove the field dirt, stones and metals, dried at 105°C until the weight was constant, and stored in desiccate before use. Analysis of the corn stover sample according to the procedures of the National Renewable Energy Laboratory revealed a composition (dry weight basis) of 37.9% glucan, 20.1% xylan, 2.3% arabinan, and 20.8% lignin.

Corn stover was pretreated by the EmimOAc–NMP solution [33]. Briefly, to a solution of EmimOAc (60 g) and NMP (140 g) preheated in an oil bath at 140°C was added corn stover sample (20 g). The suspension was held at the same temperature with stirring for 1 h, until a viscous black yellow solution formed. The solution was cooled down to 50°C, and methanol (500 mL) was added with vigorously stirring. The precipitates were filtrated, washed with methanol (500 mL) and water (2 × 500 mL), and freeze-dried to obtain regenerated corn stover samples.

### Enzymatic hydrolysis of cellulose and regenerated corn stover

Cellulose hydrolysates were made by enzymatic hydrolysis of 3.6% (w/v) sigmacell cellulose in 0.3 M phosphate buffer (pH 5.2) at 37°C, 200 rpm. Cellulase and cellobiase per gram cellulose were loaded at 7.5 FPU and 15 CBU, respectively. Corn stover hydrolysates were made by enzymatic hydrolysis of 5.0% (w/v) regenerated corn stover in 50 mM phosphate buffer (pH 4.8) at 50°C, 200 rpm. Cellulase, cellobiase and xylanase per gram dry materials were loaded at 10 FPU, 20 CBU and 10 mg, respectively.

### General procedure for the SSELP process

*C. curvatus* cells were cultivated in YPD liquid medium (yeast extract 10 g/L, peptone 10 g/L, glucose 20 g/L, pH 6.0) at 30°C, 200 rpm for 24 h. Cells were collected by centrifugation, washed with sterilized water, and then used as inocula for all two-stagedd culture processes. One unit optical absorbance at 600 nm (OD_600 nm_) for such inocula equaled to 0.36 g/L of cell dry weight (CDW). About 0.27 g of CDW equivalent inocula were transferred into a suspension of hydrolytic enzymes and 2.0 g of cellulose in 50 mL of 0.3 M phosphate buffer (pH 5.2), and other experimental details are summarized in Table 2.

### Time course of lipid production with different culture processes

A two-stagedd lipid production process was carried out at 30°C, 200 rpm. About 0.27 g of CDW equivalent inocula were resuspended in 50 mL of 0.05 M phosphate buffer (pH 5.5) contained 2.08 g glucose · H_2_O and 1% (V/V) a trace element solution [14]. The composition of the trace element solution contained (g/L): CaCl_2_^.^2H_2_O 4.0, FeSO_4_^.^7H_2_O 0.55, citric acid^.^H_2_O 0.52, ZnSO_4_^.^7H_2_O 0.10, MnSO_4_^.^H_2_O 0.076 and 100 uL of 18 M H_2_SO_4_.

For the SSELP process, 0.27 g of CDW equivalent inocula were transferred into a suspension contained 2.0 g of cellulose, 15 FPU cellulase and 30 CBU cellobiase in 50 mL of 0.3 M KH_2_PO_4_-Na_2_HPO_4_ buffer (pH 5.2), and the suspension was held at 37°C, 200 rpm for lipid production.

### Lipid production from regenerated corn stover

Experiments were first done with the SHELP process. To 50 mL of the corn stover hydrolysates contained 31.9 g/L of glucose and xylose was inoculated with 0.27 g of CDW equivalent inocula, and the culture was held at 30°C, 200 rpm for lipid production.

For the SSELP process, 0.27 g of CDW equivalent inocula were transferred into a suspension contained 2.5 g of regenerated corn stover, 10 FPU cellulase, 20 CBU cellobiase and 12.5 mg xylanase in 50 mL of 0.3 M KH_2_PO_4_-Na_2_HPO_4_ buffer (pH 5.2), and the suspension was held at 37°C, 200 rpm for lipid production.

### Analytic methods

Glucose was determined using an SBA-50B glucose analyzer (Shandong Academy of Sciences, Jinan, China). Sugar mixtures were analyzed by ion chromatography (IC) on the Dionex ICS2500 system with a CarboPac PA10 analytical column (Dionex Co.) and an ED50 electrochemical detector (Dionex Co.). The column was washed with isocratic elution of NaOH at a speed of 1 mL/min at 30°C. The concentration of NaOH was 22 mM from 0 min to 20 min, retention time for glucose and xylose were 10.9 and 12.1 min, respectively. Cellulose concentration was determined as described [34]. Residual cellulose was collected by repeated precipitation and washing with water to remove soluble carbohydrates. Precipitated sample was further washed using acetic acid-nitric acid reagent and water to remove non-cellulosic materials [35] and quantified by using the phenol-sulfuric acid method with glucose as the standard [36]. Cellulose conversion was calculated based on the initial cellulose and residual cellulose.

Samples containing cellulose and yeast cells from 30 mL of culture broth were collected by centrifugation and washed twice with distilled water. Cell mass, expressed as CDW, was determined gravimetrically after drying the wet sample at 105°C overnight and deducting cellulose from the sample. Fat-free cell mass was calculated after subtraction of lipids from CDW.

Dried samples containing cellulose and yeast cells were digested with 4 M HCl at 78°C for 1 h before extraction with chloroform/methanol (1: 1, vol/vol). The extracts were washed with 0.1% NaCl, dried over anhydrous Na_2_SO_4_, evaporated in vacuo, and the residue was dried at 105°C for 24 h to give the total lipids [37]. Lipid content was expressed as gram lipids per gram CDW. Lipid coefficient was expressed as gram lipids produced per gram cellulose.

The fatty acid compositional profiles of lipid samples were determined using a 7890F gas chromatography instrument after transmethylation according to a published procedure with minor modifications [3]. Briefly, 70 mg of lipids were treated with 0.5 mL of 5% KOH solution in methanol at 65°C for 50 min, followed by the addition of 0.2 mL BF_3_ diethyletherate and 0.5 ml methanol. The mixture was refluxed for 10 min, cooled, and extracted with n-hexane. The organic layer was washed twice with distilled water, and used for fatty acid compositional analysis.

All data in this study were the averages of three independent experiments.

## Abbreviations

CBU: Cellobiose unit; CDW: Cell dry weight; EmimOAc: 1-Ethyl-3-methylimidazolium acetate; FPU: Filter paper unit; IC: Ion chromatography; NMP: N-methylpyrrolidone; SFA: Saturated fatty acid; SHLP: Separated hydrolysis and lipid production; SHELP: Separated hydrolysis and enhanced lipid production; SSLP: Simultaneous saccharification and lipid production; SSELP: Simultaneous saccharification and enhanced lipid production; YPD: Yeast peptone dextrose

## Competing interests

The authors declare that they have no competing interests.

## Authors’ contributions

ZWG performed the experiments, analyzed the results and drafted the manuscript. HWS and XBY participated in experiments. QW participated in sugar analysis. HBX participated in biomass pretreatment and commented on the manuscript. ZKZ coordinated the study and revised the manuscript. All authors approved the final manuscript.
